# ﻿Morphology and distribution of the Middle Asian centipede genus *Krateraspis* Lignau, 1929 (Chilopoda, Geophilomorpha, Mecistocephalidae)

**DOI:** 10.3897/zookeys.1095.80806

**Published:** 2022-04-14

**Authors:** Yurii V. Dyachkov, Lucio Bonato

**Affiliations:** 1 Altai State University, Lenin Avenue 61, Barnaul 656049, Russia Altai State University Barnaul Russia; 2 Dipartimento di Biologia, Università di Padova, via U. Bassi 58b, I-35131 Padova, Italy Università di Padova Padova Italy

**Keywords:** Kazakhstan, *Krateraspis*, Kyrgyzstan, Tajikistan, taxonomy, Uzbekistan

## Abstract

A comprehensive redescription of the poorly known mecistocephalid genus *Krateraspis* Lignau, 1929 and its two species is provided, based on the examination of type material and new specimens, as well as the critical evaluation of all published information. *Krateraspis* is confirmed differing from all other Mecistocephalidae especially for a peculiar pattern of areolation and setation of the clypeus. Records from 24 localities indicate that *Krateraspis* is limited to a narrow area of Middle Asia, from the Western Tian-Shan to the western offshoots of Pamir Mountains. Two species are morphologically distinguishable: *K.meinerti* (Sseliwanoff, 1881) and *K.sselivanovi* Titova, 1975. They differ mainly in details of the clypeus and maxillae, in the pattern of forcipular denticles, and in the number of legs. *Tygarrupasiaticus* Verhoeff, 1930 is confirmed as a junior synonym of *K.meinerti*, and a lectotype is designated for the former.

## ﻿Introduction

The genus *Krateraspis* Lignau, 1929 is one of the least known and least distinct genera of the centipede family Mecistocephalidae Bollman, 1893 (Bonato et al. 2003). All reliable records are from a narrow area in the Middle Asia (Titova 1975; Dyachkov 2019, 2020; Dyachkov and Nedoev 2021), and they are currently referred to two species, *K.meinerti* (Sseliwanoff, 1881) and *K.sselivanovi* Titova, 1975 (Bonato et al. 2016). A satisfactory diagnosis of the genus is missing, the published accounts on its morphology are incomplete and contained ambiguous details, and the differential characters of the species have not been scrutinized carefully.

The first specimen of *Krateraspis* was reported by Sseliwanoff (1881a, 1881b) from near Tashkent (Uzbekistan) and was originally described as a species of *Mecistocephalus* Newport, 1843, namely *M.meinerti*, with very incomplete morphological information and without illustrations. Other specimens collected later near the type locality allowed Lignau (1929a, 1929b) and Verhoeff (1930) to complement the morphological description of this species and to provide the first illustrations. However, while Lignau (1929a, 1929b) assigned his specimen to *M.meinerti* and separated the species in a distinct genus *Krateraspis*, Verhoeff (1930) described his specimens as a new species of *Tygarrup* Chamberlin, 1914, namely *T.asiaticus*. The latter name was recognized as a synonym of *Krateraspismeinerti* by Titova (1975). In the same paper, Titova described a second species of *Krateraspis*, namely *K.sselivanovi* Titova, 1975, from Sharak (Tajikistan), as well as a putative third species from the Russian Far East, namely *K.striganovae* Titova, 1975, which however has been later recognized in a distinct genus *Agnostrup* Foddai, Bonato, Pereira & Minelli, 2003. Other records of *Krateraspismeinerti*, additional information on its morphology and some first photographs were published by Dyachkov (2019, 2020) and Dyachkov and Nedoev (2021).

This paper contributes a comprehensive redescription of the morphology of the genus *Krateraspis* and its species, and an update of their distribution, based on the examination of the available type material and the critical evaluation of all published information.

## ﻿Materials and methods

We examined the holotype of *K.meinerti* (at ZISP; for abbreviations see below), one of the syntypes of its junior synonym *Tygarrupasiaticus* (at NHRS), at least six paratypes and some other possible paratypes of *K.sselivanovi* (at ZMMU; see corresponding Remarks), a specimen originally assumed by Verhoeff (1930) to be a juvenile *T.asiaticus* (at ZMB), 67 specimens of *K.meinerti* (at ASU, ZMMU, and ZISP) already reported by Dyachkov (2019, 2020) and Dyachkov and Nedoev (2021), and 18 other specimens of *K.meinerti* and nine of *K.sselivanovi* (at ZMMU).

The specimens were examined with stereo microscopes: Olympus SZX16, Olympus BX51, Leica Z16 APO. Some non-typical specimens of *K.meinerti* and *K.sselivanovi* were dissected, and their cephalic capsule, forcipular segment, mandibles, maxillary complex, and remaining body were mounted in permanent slides using euparal. Photographs were taken using an Olympus DP74 or a Leica DFC490 digital cameras attached to the microscopes. Measurements were taken from the photos using the software FAST 1.0 (Vaganov et al. 2020).

We compiled a revised diagnosis of the genus *Krateraspis* by comparison with all currently recognized genera of Mecistocephalidae (Bonato et al. 2003; Uliana et al. 2007; Bonato et al. 2016). We also revised the differential diagnoses between the species of *Krateraspis* by direct comparison of specimens and critical reinterpretation of the published accounts. The terminology of morphology follows Bonato et al. (2010b).

Localities are indicated as in the original labels or publications. Modern English names and additional information are in square brackets. All localities were georeferenced unambiguously, with the single exception of “Fayzabad” because there are at least two homonymous villages with this name (Dyachkov 2020: 78). Localities were mapped with SimpleMappr (Shorthouse 2010).

### ﻿Abbreviations

**AF** A.A. Fomichev;

**AR** A. Ryvkin;

**ASU** Altai State University (Barnaul, Russia);

**D** Daniyarov;

**dors.** dorsal.;

**juv.** juvenile/s;

**LB** L. Berg;

**LBS** leg-bearing segment(s);

**lg** legit;

**MG** M.S. Ghilarov;

**NHMUK**Natural History Museum, London;

**NHMW** Natural History Museum, Vienna;

**NHRS**Swedish Museum of Natural History, Stockholm;

**NZ** N.A. Zarudniy;

**Tj** Tajikistan;

**V** Veltishev;

**ventr.** ventral;

**VR** V. Russov;

**YD** Yu.V. Dyachkov;

**ZISP**Zoological Institute of the Russian Academy of Sciences, Saint Petersburg;

**ZMB**Museum für Naturkunde, Berlin;

**ZMMU**Zoological Museum of the Moscow State University;

**ZSM**Zoologische Staatssammlung, München.

## ﻿Results

### ﻿ Krateraspis

Taxon classificationAnimaliaGeophilomorphaMecistocephalidae

Lignau, 1929

3EBBAB70-00CD-5A50-9EFB-F1789FD5D14D

Krateraspis : Lignau 1929a: 160 (available name), 165. Lignau 1929b: 207 (original description). Verhoeff 1930: 265. Titova 1975: 39, 46 (in key). Titova 1983: 148. Bonato et al. 2003: 544, 547, 549, 550, 552, 553. Foddai et al. 2003: 1255. Bonato et al. 2009: 195, 199, 207. Bonato et al. 2010a: 515. Bonato and Zapparoli 2011: 331. Bonato 2011: 434. Volkova 2016: 675. Dyachkov 2019: 368, 370, 372. Dyachkov 2020: 79; Dyachkov and Nedoev 2021: 44.

#### Type species.

*Mecistocephalusmeinerti* Sseliwanoff, 1881, by monotypy (Lignau 1929a, 1929b).

#### Remarks on nomenclatural issues.

The genus name *Krateraspis* was first introduced by Lignau (1929a) without a description or diagnosis, but it was explicitly used for the species *Mecistocephalusmeinerti* Sseliwanoff, 1881 and therefore it is available since that publication (ICZN 1999: Art. 12.2.5). Instead, the first morphological description of *Krateraspis* was given in a different paper by the same author, published in the same year but in a later date (Lignau 1929b).

The type species of *Krateraspis* was determined by monotypy (see also Jeekel 2005: 86), not by original designation as erroneously reported by Bonato et al. (2016) and Dyachkov (2019).

#### Diagnosis.

A genus of Mecistocephalidae with: anterior areolate part of the clypeus extending along the lateral margins of the clypeus to the labrum; two clypeal plagulae separated by a mid-longitudinal areolate strip; central part of the clypeus with distinct but fainter areolation in comparison with the markedly areolate anterior part and the mid-longitudinal strip; clypeal setae only three or four pairs, on the antero-central part of the clypeus; buccae without spiculum; labral anterior ala with the internal margin reduced to a point; labral posterior ala with the posterior margin entire, without bristles; coxosternite of first maxillae divided by a mid-longitudinal suture; coxosternite of second maxillae entire, without mid-longitudinal suture, with the grooves from the metameric pores reaching the lateral margins of the coxosternite at approximately their mid-length; telopodite of second maxillae bearing a small claw-like pretarsus; forcipular tergite slightly wider than long; sternites without pore fields; either 45 or 53 pairs of legs; ultimate legs without claw but with an apical small spine.

*Krateraspis* differs from other mecistocephalids (Table 1) mainly in the pattern of clypeal areolation and setation: a broad weakly areolate central part of the clypeus is distinguishable from the distinctly areolate anterior part as well as the non-areolate posterior plagulae, and a few setae are present on the medial part only. Of two other Middle Asian mecistocephalid genera, *Tygarrup* and *Arrup*, *Krateraspis* is more similar to the former. *Tygarrup* differs from *Krateraspis* for an entire non-areolate plagula lacking a mid-longitudinal areolate strip, and for the presence of setae on both the central and lateral parts of the clypeus. *Arrup* differs from *Krateraspis* not only in the clypeus (markedly areolate in both the central and anterior part, with setae on the both the lateral and central parts), but also in the maxillary complex (coxosternite of the first maxillae entire, without mid-longitudinal suture; coxosternite of the second maxillae with grooves from the metameric pores running backwards towards the posterior corners of the coxosternite), in the forcipular tergite (much wider than long), and the number of legs (41 pairs).

#### Included species.

*Krateraspismeinerti* (Sseliwanoff, 1881) and *K.sselivanovi* Titova, 1975.

#### Distribution.

**Figure 1.**
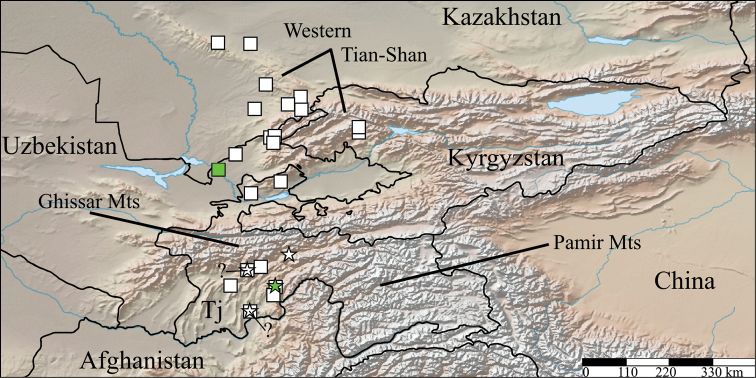
Distribution of *Krateraspis* Lignau, 1929: square, *K.meinerti* (Sseliwanoff, 1881); star, *K.sselivanovi* Titova, 1975. Green symbols indicate type localities. Some very close localities are marked by a single symbol. The question marks indicate alternative positions of the uncertain locality “Fayzabad” (see Materials and methods).

Recorded from 24 localities in Middle Asia so far, from Western Tian-Shan to the western offshoots of Pamir Mts (Fig. 1).

#### Remarks on published morphological accounts.

**Figures 2–9.**
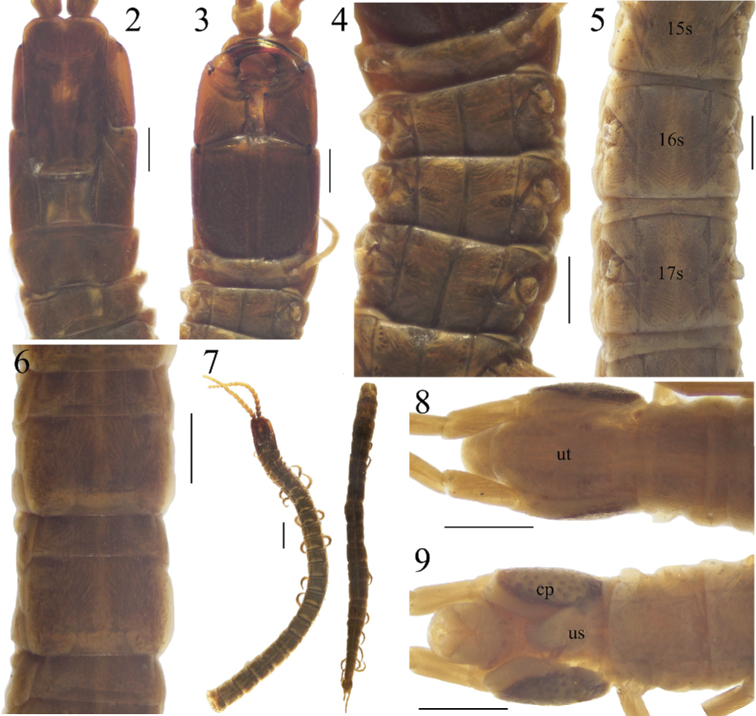
Holotype of *Krateraspismeinerti* (Sseliwanoff, 1881), from Chinaz near Tashkent (ZISP): **2, 3** head, forcipular and LBS 1 (dors., ventr.) **4** anterior LBS (ventr.) **5, 6** intermediate LBS (ventr., dors.) **7** anterior and posterior parts of the body (dors.) **8, 9** terminal part of the body (dors., ventr.). Abbreviations: 15s, 16s, 17s – metasternites 15–17, cp – coxopleural pores, us – metasternite of the ultimate LBS, ut – metatergite of the ultimate LBS. Scale bars: 0.5 mm (**2–6, 8–9**); 2 mm (**7**).

**Figures 10–14.**
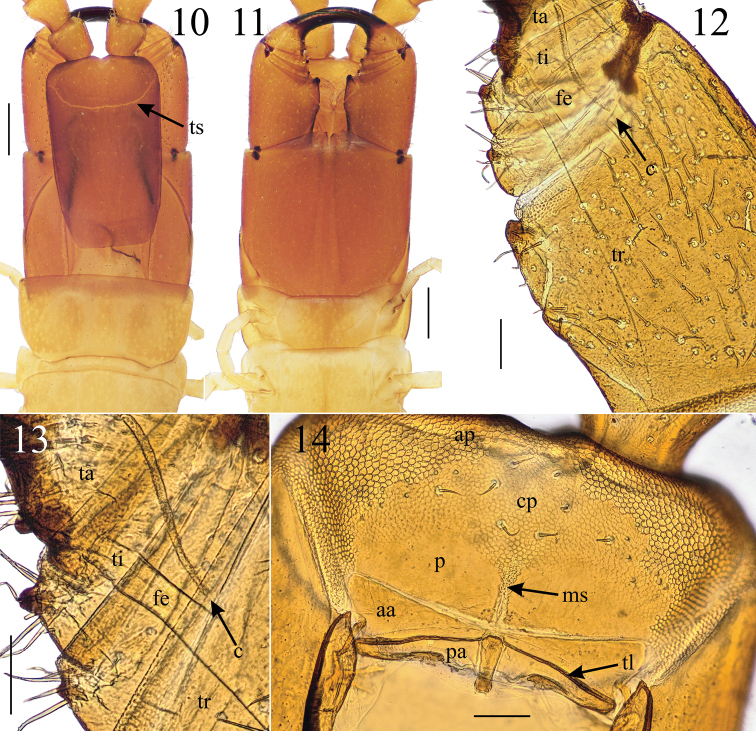
*Krateraspismeinerti* (Sseliwanoff, 1881): **10, 11** head, forcipular and LBS 1 (dors., ventr.) **12, 13** left forcipule (ventr.) **14** clypeus and labrum (ventr.). Specimens: ♀ (**10, 11, 13, 14**) and ♂ (**12**), from Syrdarya-Turkestan Natural Park (ASU No. 216). Abbreviations: aa – anterior ala, ap – markedly areolate anterior part of clypeus, c – calyx of poison gland; cp – central part of clypeus with distinct but fainter areolation, fe – femur, ms – mid-longitudinal areolate strip, p – plagula, pa – posterior ala, ta – tarsungulum, ti – tibia, tl – transverse thickened line, tr – trochanteroprefemur, ts – transverse suture. Scale bars: 0.5 mm (**10, 11**); 0.1 mm (**12–14**).

The peculiar pattern of clypeal areolation is well recognizable only using a light microscope with slides, while it is very poorly visible using stereo (dissecting) microscope. Additionally, the semblance of the areolation is conditioned by the preparation of the specimen, the optical properties of the inclusion medium and the mode of illumination. This may explain why the pattern of areolation on the clypeus has been interpreted, described, and illustrated in inconsistent ways by different authors. Lignau (1929b) did not distinguish between a markedly areolate anterior part and a weakly areolate central part, neither in the textual description of *K.meinerti* (“Vorderklypeus fein gefeldert, nimmt etwas mehr als die Hälfte der gesammten Fläche ein” [anterior clypeus finely areolate, extending a little more than half of the total area]) nor in the associated illustration (his fig. 10). In the same way, Titova (1975) described *K.sselivanovi* without indicating any variation in the areolation between anterior and central parts of the clypeus, neither in the textual description (“Peredniy clypeus zanimaet bolee poloviny nalichnika, ego poverhnost sostoit iz polygonalnikh poley, po seredine uzkoy polosoy razdelyayuschikh zadniy clypeus na 2 poloviny” [anterior clypeus covers more than a half of the total clypeal area, its surface consists of polygonal cells that divide the posterior clypeus in the middle into 2 parts by a narrow strip], nor in the accompanying illustration (her fig. 2: 1A). On the other hand, Verhoeff (1930) described and illustrated *T.asiaticus* (synonym of *K.meinerti*, see below) ignoring the weak areolation in the central part of clypeus and assigning this part to the non-areolate plagulae. Dyachkov (2019) used term “insula” for the weakly areolate central part of the clypeus of *K.meinerti*, but the term was previously used for a non-areolate area inside the areolate anterior clypeus (Bonato et al. 2010b).

**Table 1.** Main differences between *Krateraspis* and the other genera of Mecistocephalidae.

Characters	*Krateraspis* Lignau, 1929	*Arrup* Chamberlin, 1912	*Partygarrupius* Verhoeff, 1939	*Agnostrup* Foddai, Bonato, Pereira & Minelli, 2003	*Nannarrup* Foddai, Bonato, Pereira & Minelli, 2003	*Dicellophilus* Cook, 1896	*Anarrup* Chamberlin, 1920	*Proterotaiwanella* Bonato, Foddai & Minelli, 2002	*Tygarrup* Chamberlin, 1914	*Mecistocephalus* Newport, 1843	*Takashimaia* Miyosi, 1955
clypeus: central part: areolation	yes	yes	yes	yes	yes	no	no	yes	no	yes	no
clypeus: areolation of central part compared with anterior part	fainter	similar	similar	similar	similar	no	no	similar	no	similar or fainter; sometimes non-areolate insulae	no
clypeus: posterior mid-longitudinal areolate strip	yes	yes	no	yes	yes	no	no	yes	no	yes	no
clypeus: areolation extending all along the lateral margins	yes	yes	no	no	yes	no	no	yes	no	no	no
clypeus: setae on antero-lateral corners	no	yes	no	no	no	yes	yes	no	usually yes	yes or no	no
clypeus: setae on lateral parts	no	longitudinally elongate areas	narrow transverse band	longitudinally elongate areas	longitudinally elongate areas	longitudinally elongate areas	narrow transverse band	longitudinally elongate areas	narrow transverse band	narrow transverse band	no
bucca: spiculum	no	no	no	no	no	no	no	no	no	yes	yes
labrum: anterior ala: internal margin reduced to a point	yes	yes	yes	yes	yes	yes	yes	yes	yes	no	yes
labrum: posterior ala: posterior margin: bristles	no	no	no	no	no	yes	no	no	no	usually no	no
first maxillae: coxosternite: mid-longitudinal suture	yes	no	yes	yes	yes	yes	yes	yes	yes	yes	yes
second maxillae: coxosternite: mid-longitudinal suture	no	no	no	no	no	no	yes	no	no	no	no
second maxillae: coxosternite: groove from metameric pore reaching lateral margin	yes	no	no	no	no	no	yes	yes	yes	yes	yes
second maxillary telopodite: distinctly surpassing the first maxillary telopodite	yes or no	no	no	no	no	yes	yes	no	yes	yes	yes
second maxillae: pretarsus	small claw	no or small claw	small claw	no	no	spinous tubercle	spinous tubercle	small claw	small claw	small claw	small claw
forcipular segment: tergite: width/length	~1.5	~2.0	~1.5	~2.0	~2.0	~1.5	~1.5	~1.5	~1.5	~1.5	~1.5
trunk: leg-bearing segments	45 or 53	41	41	41	41	41 or 43 or 45	41	45 or 49	43 or 45	45 or more	45
ultimate leg pair: pretarsus	no	no	no	no	no	spinous tubercle	spinous tubercle	spinous tubercle	no	no	no

The pattern of clypeal setae and sensilla has also been reported inconsistently: the eight “Punkte” [points] described and illustrated by Lignau (1929b: fig. 10) in the central part of clypeus of his single specimen of *K.meinerti* are probably the sockets of broken setae, because these points (in his fig. 10) correspond in number and position with the eight clypeal setae present in most specimens of this species. In the same way, Lignau (1929b) described the antennae as “kahl” [without setae] probably because the antennal setae were broken in his material.

The description of *K.meinerti* provided by Lignau (1929b) includes another obvious mistake: the sentence “2. Maxille mit getrennten Hüften” [second maxillae with divided coxosternite] should be read “1. Maxille mit getrennten Hüften” [first maxillae with divided coxosternite], because it is contradicted by a previous sentence in the same text (“ganz verwachsenen Hüften der 2. Maxille” [entirely coalescent coxosternite of the second maxillae”], as well as by an associated illustration (his fig. 9).

### ﻿ Krateraspis meinerti

Taxon classificationAnimaliaGeophilomorphaMecistocephalidae

(Sseliwanoff, 1881)

53DF60FF-0AE8-57C9-9893-B626B3449173

Figures 2–9 , 10–14 , 15–17 , 18–24 , 25–28

Mecistocephalus meinerti : Sseliwanoff 1881a: 9 (nomen nudum). Sseliwanoff 1881b: 232 (original description). Sseliwanoff 1884: 73 (description). Attems 1903: 168, 210. Attems 1904: 115. Attems 1914: 21. Attems 1929: 156. Izotova 1960: 150 (misidentification).
Krateraspis meinerti : Lignau 1929a: 160, 165 (new record). Lignau 1929b: 207 (redescription); figs 7–11. Verhoeff 1930: 264. Titova 1965: 871 (new record). Titova 1969: 165. Titova 1975: 39, 45 (new records), 46 (in key); fig. 2: 2–4B. Bonato et al. 2003: 543, 545, 546, 550, 551, 577. Ilie et al. 2009: 14. Bonato 2011: 434. Volkova 2016: 675. Dyachkov 2019: 368, 371 (new records; description), 373 (in key); figs 6–10. Dyachkov 2020: 79 (new records), 85. Dyachkov and Nedoev 2021: 44 (new records), 47.
Tygarrup asiaticus : Verhoeff 1930: 260 (original description); figs 20–21. Verhoeff 1934: 31. Verhoeff 1937: 235 (in key). Verhoeff 1939: 88 (in key). Takakuwa 1940: 84. Verhoeff 1940: 31. Verhoeff 1942: 49 (in key). Shinohara 1965: 303 (in key), 304. Titova 1965: 871, 874 (in key). Titova 1983: 147, 148.

#### Type locality.

“Chinas, bl. Tashkenta” (Sseliwanoff 1881a), also indicated as “Chinad [*sic*] bliz Tashkenta” (Sseliwanoff 1881b) and “Mestechko Chinas, bliz Tashkenta” (Sseliwanoff 1884) [Uzbekistan, Tashkent region, Chinaz town, ca. 40°56'N, 68°45'E].

#### Synonyms.

*Tygarrupasiaticus* Verhoeff, 1930 (synonymization since Titova 1975; see below, under Remarks).

#### Examined specimens.

***Holotype*** of *Mecistocephalusmeinerti* Sseliwanoff, 1881: ♀, from Chinas bl. Tashkenta [Uzbekistan, Tashkent region, Chinaz near Tashkent], 1878, VRlg (ZISP). ***Lectotype*** of *Tygarrupasiaticus* Verhoeff, 1930 (see below, under Remarks): ♂, from Tashkent (NHRS-JONI 714). ***Other material***: 1 ♀, from Tashkent, 13.III [year unknown], NZlg (ZISP chilo-52); 1 ♀, from Ugam Mts, Sidzhak, nut [*Juglans*] forest, soil samples, 28.IV.[19]74, MGlg (ZMMU Rc 7408); 1 ♀, from Chimgan, nut forest, 07.V.[19]74, MGlg (ZMMU Rc 7413); 1 ♀, from Chimgan, Tashkent ravine, VII.[19]06, LBlg (ZISP chilo-1); 1 ♀, from Vrevskaya Station [now Almazar, ca. 40°57'N, 68°50'E], 26.IV.1932, V lg (ZISP chilo-5); 1 ♂ and 1 ♀, from Kamsay, near Khumsan, *Juglandetum*, 03.V.[19]74, MGlg (ZMMU Rc 7407); 1 ♂ and 5 ♀♀, from Khumsan, right bank of Ugam river, nut forest, 1.V.[19]74, MGlg (ZMMU Rc 7406); 1 ♀, from [Tajikistan, Districts of Republican Subordination, Roghun district] left side of Obi­kandak river valley (left stream tributary of Obigarm river), stony meadow with rocks, 38°43.275'N, 69°43.863'E, 1250–1540 m, 23.IV.2019, AFlg (ASU No. 261); 2 ♂♂, from [Khatlon region, Mu’minobod district], Muminabad [Mu’minobod, now Leningradsky, ca. 38°06'N, 70°01'E], 0–10 [cm deep], 19.V.[19]62 (ZMMU Rc 8158) and 11.V.[19]65 (ZMMU Rc 8159); 2 ♂♂, 3 ♀♀ and 1 body fragment from Sharak [village, ca. 38°16'N, 70°04'E], 10–20 [cm deep], 15.VIII.[19]65 (ZMMU Rc 8136), 10–20 [cm deep], 27.V.[19]65 (ZMMU Rc 8185), 20–30 [cm deep], 19.X.[19]64 (ZMMU Rc 8148), 10–20 [cm deep], 31.V.[19]65 (ZMMU Rc 8155), grass, 0–10 [cm deep], 3.VI.[19]63 (ZMMU Rc 8139); 1 ♂ and 6 ♀♀, from [Yovon district], Yavan [Yovon, ca. 38°18'N, 69°03'E]: *Triticum*, 20–40 cm deep, 25.VII.[19]67 (ZMMU Rc 8151), *Triticum*, 20–60 [cm deep], 20.X.[19]67 (ZMMU Rc 8186), *Triticum*, 0–30 [cm deep], 19.X.[19]68 (ZMMU Rc 8170), *Triticum*, 10–20 [cm deep], 21.V.[19]68 (ZMMU Rc 8183), *Hordeum*, 0–10 [cm deep], 13.V.[19]67 (ZMMU Rc 8169), *Avena*, 0–10 [cm deep], 26.V.[19]68 (ZMMU Rc 8172); 2 ♂♂ and 4 body fragments, from [Sughd region], Matcha district [ca. 40°32'N, 69°25'E]: 10–20 [cm deep], [date unknown], D lg (ZMMU Rc 8157), 15.V.[19]65 (ZMMU Rc 8149), and 0–10 [cm deep], D lg (ZMMU Rc 8190); 1 ♀, from Mogol-Tau Mts [ca. 40°23'N, 69°31'E], under stones, [19]74 (ZMMU Rc 7409); 1 ♀, from F-bad [unknown region, Fayzobod], *Triticum*, 10–20 [cm deep], 6.V.[19]66 (ZMMU Rc 8174); 1 ♂, from [Kyrgyzstan, Jalal-Abad region], Sary-Chelek Nature Reserve, near Arkit Village [ca. 41°47'N, 71°57'E], forest with *Juglans* and *Acer*, 03.VII.[19]83, ARlg (ZMMU Rc 7670); 1 ♂, from near Kyttelsay stream, forest with *Juglans*, 04.VII.[19]83, ARlg (ZMMU Rc 7667); 5 ♂♂, 3 ♀♀ and 3 juv., from Kazakhstan, Turkistan region, 10 km SW Abay Village, Karatau Mt. Range, Karatau State Nature Reserve, cereals and tulip steppe, under stones, 43°47'04.2"N, 68°46'42.0"E, 1020 m, 06–07.V.2017, YDlg (ASU No. 214); 1 ♀, from 50 km NW Achisay Village, Kyzylkol Lake coast, in clay stones, 43°46'34.0"N, 69°30'36.4"E, 328 m, 08–09.V.2017, YDlg (ASU No. 215); 5 ♂♂, 10 ♀♀ and 5 juv., from Karatau Mt Range, Syrdarya-Turkestan Natural Park, near Terekty Village, Boralday River coast, *Morus* and cereals, under stones, 42°51'48.2"N, 69°51'55.0"E, 529 m, 14–15.V.2017, YDlg (ASU No. 216); 9 ♂♂, 6 ♀♀ and 3 juv., from Ugam Mt Range, Sayram-Ugam National Park, 10 km NE Tylkubas Village, Iirsu River Valley, meadow, under stones, 42°24'58.0"N, 70°21'30.08"E, 1296 m, 16–18.V.2017, YDlg (ASU No. 217).

#### Remarks on nomenclatural issues.

The species name *Mecistocephalusmeinerti* was first introduced by Sseliwanoff (1881a) without description, definition, or indication, and therefore it is not available from that publication (ICZN 1999: Art. 12.1 and 12.2). The name became available since another paper published later (Sseliwanoff 1881b), which provided a morphological description of the species, based on a specimen.

Verhoeff (1930) described *Tygarrupasiaticus* based on nine specimens from two localities (seven from Vreskaja, ca. 50 km SW of Tashkent, and two from Tashkent) and all these specimens should be considered as syntypes (ICZN 1999: Recommendation 73F). They are preserved in different museums: at least four in ZSM (SysTax 2021), one in ZMB (Moritz and Fischer 1979; pers. obs.), one in NHMUK (Natural History Museum 2021), one in NHRS (pers. obs.), and one in NHMW (Ilie et al. 2009). The descriptions and illustrations provided by Verhoeff and our direct examination of two syntypes (NHRS-JONI 714 and ZMB 3610) revealed that Verhoeff described *T.asiaticus* mainly on some syntypes that are fully consistent with *Krateraspismeinerti*. Other syntypes actually belonging to another species were misinterpreted by Verhoeff as juveniles of *Tygarrupasiaticus*. To stabilize the usage of the name, we herewith designate NHRS-JONI 714 as lectotype of *T.asiaticus* (ICZN 1999: Art. 74.1.1). This specimen (Fig. 18) is fully consistent with the original description and illustrations published by Verhoeff (1930) for the adult morphology of *T.asiaticus* and *Krateraspismeinerti*. It is an adult male 31 mm long, labeled “*Tygarrupasiaticus* Verh. Turkestan”, acquired by NHRS in 1931 and indicated explicitly as type in the catalogue of NHRS. This specimen has been now labeled “lectotype”, whereas other previous syntypes has been now labeled “paralectotype” (ICZN 1999: Recommendation 74C).

ZMB 3610 (labeled as a syntype of *T.asiaticus*, from Tashkent, with 43 pairs of legs; Figs 29–32) actually belongs to a species of *Arrup* Chamberlin, 1912, as indicated by the following characters: clypeus with many setae on the lateral parts and very short paired plagulae (Fig. 30), first maxillae with relatively small telopodites (Fig. 30), forcipular tarsungulum with a relatively long denticle (Fig. 32), and 41 pairs of legs (erroneously reported 43 on the label on the microscopic slide; Fig. 29). More precisely, ZMB 3610 probably belongs to the species *A.asiaticus* (Titova, 1975), which is already known from Middle Asia and differs from all other known species of *Arrup* in the variable presence of coxal organs and pores (apparently absent in some specimens, including well grown specimens) and the branching structure of the channels of the anal organs and their broad openings (Fig. 31; Titova 1975; Dyachkov 2019).

#### Diagnosis.

A species of *Krateraspis* with: clypeus showing the transition between marked and weak areolation very close to the clypeal anterior margin (at ca. 0.1 of the medial length of the clypeus), so that all clypeal setae are inside the weakly areolate central part of the clypeus; some small spine-like sensilla on the lateral parts of the clypeus; second maxillary telopodites distinctly surpassing the tips of the telopodites of the first maxillae; first article of the second maxillary telopodites without a distinct distal bulge on the external side; all forcipular articles with a distinct denticle; invariably 45 pairs of legs. See also Table 2.

**Table 2.** Main differences between *Krateraspismeinerti* (Sseliwanoff, 1881) and *K.sselivanovi* Titova, 1975.

Morphological characters	*K.meinerti*	*K.sselivanovi*
Clypeus: transition between marked and weak areolation: longitudinal position	very close to the anterior margin of the clypeus	at ca. 0.3–0.4 of the total length of the clypeus
First maxillae: telopodite: first article: distal bulge on external side	absent	present
Second maxillae: telopodite: elongation	distinctly surpassing the tip of first maxillary telopodite	approximately reaching the tip of first maxillary telopodite
Forcipule: femur: denticle	yes	no
Leg-bearing segments: number	45	53

#### Redescription of holotype.

Body stiffened, divided in two parts (Fig. 7); many legs missing. Total length ca. 48 mm; maximal width 1.4 mm (at ca. LBS 21–22). Color (in 70% ethanol) brown.

***Head*** (Fig. 2). Cephalic plate 1.7 × as long as wide, sub-rectangular but slightly widening anteriorly, its posterior margin straight. Transverse suture distinct, with a medial forward angle. Antennae ca. 5 mm, ca. 4.5 × as long as the head maximum width.

***Forcipular segment*** (Figs 2, 3). Tergite sub-trapezoid, ca. 1.5 × as wide as long, with a mid-longitudinal distinct furrow inside an oval depression. Coxosternite as long as wide, with a pair of small anterior denticles. Trochanteroprefemur 1.4 × as long as wide; tarsungulum 2.9 × as long as wide. All forcipular articles with denticles: a large distal denticle on the trochanteroprefemur, femur and tibia each with a small denticle, tarsungulum with a basal small denticle. Inner edge of tarsungulum slightly serrated.

***Leg-bearing segments*** (Figs 4–6). Tergites 2–43 with a pair of paramedian sulci. Metasternites 2–44 with a median longitudinal sulcus. 45 LBS. Legs 1 slightly smaller than following legs; pretarsi with two accessory spines.

***Ultimate leg-bearing segment*** (Figs 8, 9). Metatergite shield-shaped, 1.7 × as long as wide, and 1.2 × as wide as the pretergite. Metasternite subtriangular, 1.1 × as wide as long, its anterior margin ca. 3 × as wide as the posterior one. Ca. 50 pores on each coxopleuron, scattered on ventral and lateral sides. Legs slender, but incomplete (missing tarsus 2 of right leg, tibia and both tarsi of left leg).

**Figures 15–17.**
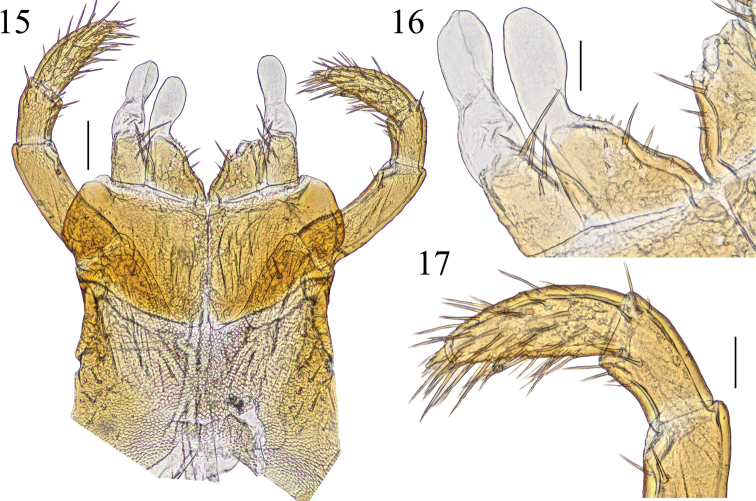
*Krateraspismeinerti* (Sseliwanoff, 1881), ventr.: **15** maxillary complex **16** right telopodite and coxal projection of first maxillae **17** left telopodite of second maxillae. Specimen: ♀ from Syrdarya-Turkestan Natural Park (ASU No. 216). Scale bars: 0.1 mm (**15**); 0.05 mm (**16, 17**).

***Postpedal segments*** (Figs 8, 9). Intermediate sternite and first genital sternite well-developed. Gonopods bi-articulate, triangular, and touching each other at their bases. Anal pores present.

#### Intraspecific variation.

Maximum body length: 71 mm in ♀♀ (*n* = 44; the largest specimen in the sample ZMMU Rc 7406), 58 mm in ♂♂ (*n* = 31). Color (in 70% ethanol) usually yellow, with cephalic plate, forcipular segment, and antennae light brown (Figs 10, 11).

***Head*.** Anterior markedly areolate part of the clypeus extending medially for 10–17% of the total clypeal length (Fig. 14). Clypeal setae usually 8, rarely 6. Labral mid-piece usually pointed and projecting backwards beyond the posterior margins of the labral lateral ones. Each mandible (Fig. 21) usually with six lamellae, with 5–9 teeth in each lamella. Second maxillae (Figs 15–17): 1^st^ article invariably without a distinct distal bulge on the external side; distal parts of 2^nd^ and 3^rd^ articles usually with numerous setae.

**Figures 18–24.**
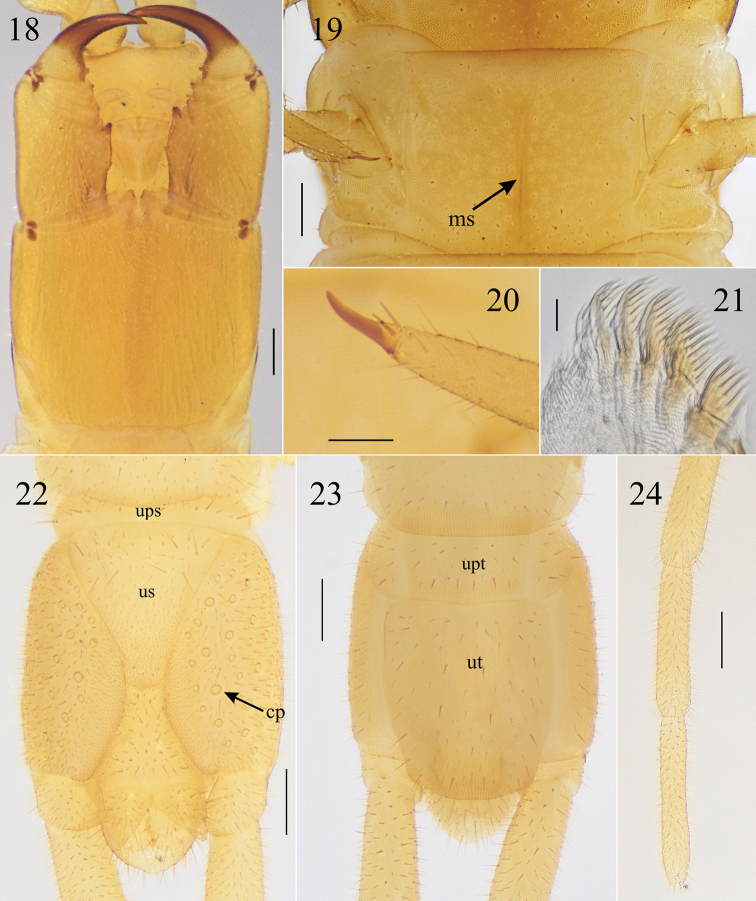
*Krateraspismeinerti* (Sseliwanoff, 1881): **18** head and forcipular segment (ventr.) **19**LBS 2 (ventr.) **20** distal end of tarsus of leg 12 (lateral view) **21** left mandible (ventr.) **22, 23** ultimate LBS and postpedal segments (ventr., dors.) **24** terminal articles of ultimate leg (ventr.). Specimens: **18** lectotype of *Tygarrupasiaticus* Verhoeff, 1930 from Tashkent (NHRS-JONI 714) **19–24** ♀ from Syrdarya-Turkestan Natural Park (ASU No. 216). Abbreviations: cp – coxopleural pores, ms – median longitudinal sulcus, ups – presternite of ultimate LBS, upt – pretergite of ultimate LBS, us – metasternite of ultimate LBS, ut – tergite of ultimate LBS. Scale bars: 0.2 mm (**18, 19, 22–24**); 0.1 mm (**20**); 0.02 mm (**21**).

***Forcipular segment*.** Tergite usually partially covered by tergite 1 (Fig. 10) and forcipules usually surpassing the anterior margin of the cephalic plate (Figs 11, 18). All forcipular articles with denticles (Figs 11–13), with the single exception of a specimen missing the denticle on the right femur (collected together with other specimens with usual morphology, in the sample ASU No. 216). Worth noting is that an analogous case of asymmetry has been detected in a specimen of *K.sselivanovi*, where a denticle has been recognized on one femur but not in the other femur (see below). Distal denticle on the trochanteroprefemur usually larger than all other denticles (Fig. 12). Denticle on the tibia slightly larger than the denticle on the femur and the basal denticle on the tarsungulum (Figs 12, 13). Calyx of poison gland usually reaching the trochanteroprefemur in both sexes (Figs 12, 13).

**Figures 25–28.**
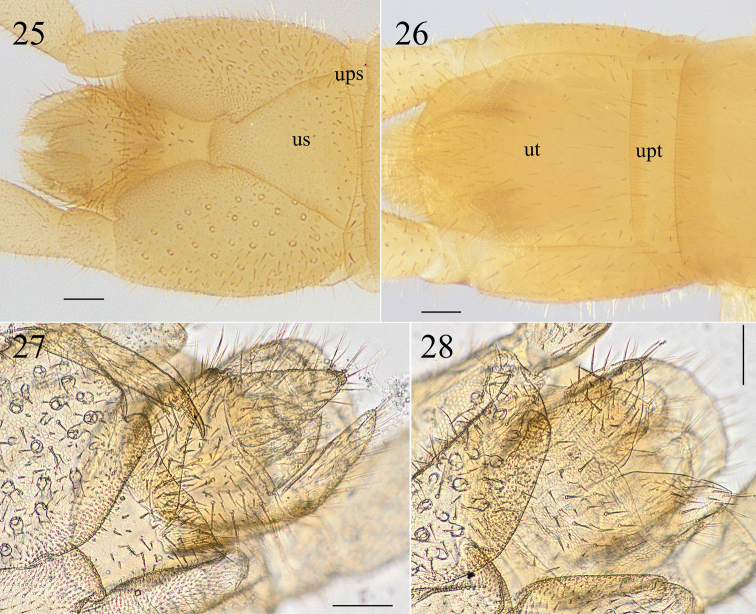
*Krateraspismeinerti* (Sseliwanoff, 1881): **25, 26** ultimate LBS and postpedal segments (ventr., dors.) **27, 28** postpedal segments (♂ and ♀; ventr.). Specimens: ♂ (**25, 26**), from Sayram-Ugam National Park (ASU No. 217); ♂ (**27**) and ♀ (**28**), from Syrdarya-Turkestan Natural Park (ASU No. 216). Abbreviations: ups – presternite of ultimate LBS, upt – pretergite of ultimate LBS, us – metasternite of ultimate LBS, ut – tergite of ultimate LBS. Scale bars: 0.1 mm.

***Leg-bearing segments*.** Invariably 45 pairs of legs. Worth noting is that *K.sselivanovi* has invariably 53 pairs of legs and the difference of eight pairs between the two species corresponds to a putative evolutionary change that have repeatedly occurred in the Mecistocephalidae (Bonato et al. 2003).

***Ultimate leg-bearing segment*.** Almost similar in both sexes, slightly thickened in male (Figs 22, 23, 25, 26). Metasternite subtriangular, its length to width ratio varying between 0.8 and 1.1, and the anterior margin 3–5 wider than the posterior one; up to ca. 50 pores on each coxopleuron in both sexes; legs densely setose, without pretarsus in both sexes.

***Postpedal segments*.** Densely setose in both sexes (Figs 22, 23, 25–28). Male gonopods bi-articulate, narrower and separated by a conic projection in between (Figs 25, 27). Female gonopods bi-articulate, subtriangular, and touching each other at their bases (Figs 22, 28).

**Figures 29–32.**
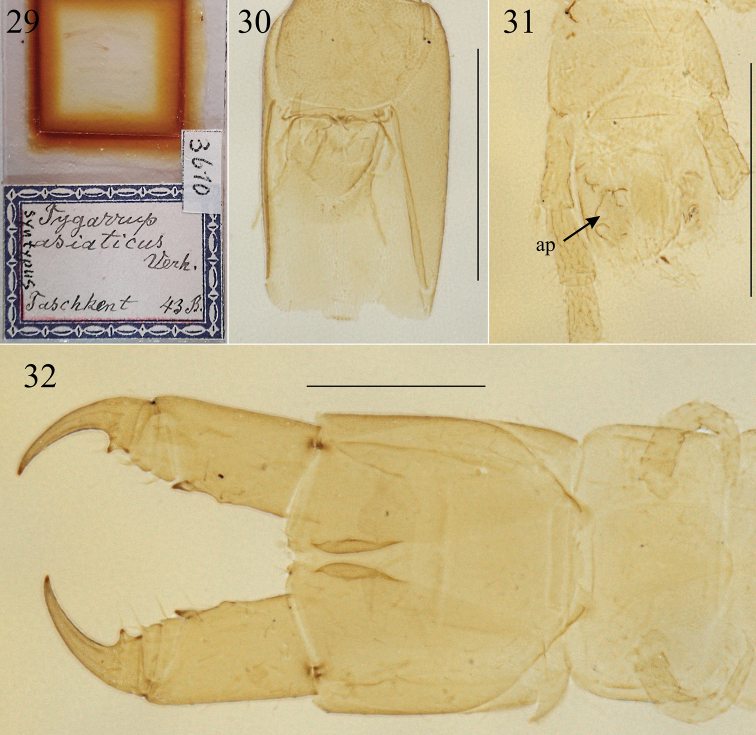
Specimen of *Arrup* misidentified by Verhoeff (1930) as juvenile *Tygarrupasiaticus* Verhoeff, 1930 (ZMB 3610), ventr.: **29** microscopic slide **30** head **31** ultimate LBS and postpedal segments; **32** forcipular segment and LBS 1. Abbreviation: ap – anal pore. Scale bars: 0.5 mm (**30–32**).

#### Distribution.

Recorded from 24 localities, from Western Tian-Shan to the western offshoots of Pamir Mts (Fig. 1), in the following countries and administrative units: Kazakhstan (Turkistan and Jambyl regions), Uzbekistan (Tashkent region), Kyrgyzstan (Jalal-Abad region), and Tajikistan (Region of Republican Subordination, Khatlon, and Sughd regions) (Sseliwanoff 1881a, 1881b, 1884; Lignau 1929a, 1929b; Titova 1965, 1975; Dyachkov 2019, 2020; Dyachkov and Nedoev 2021; present records).

A specimen from Tatarstan (European Russia) was assigned by Izotova (1960) to *K.meinerti* with doubt (see also Volkova 2016; Dyachkov 2019). The relative size of the forcipular tergite (Izotova 1960: fig. 6) shows that this specimen does not belong to Mecistocephalidae, and the shape of the forcipular segment suggests instead a species of the geophilid genus *Arctogeophilus* Attems, 1909. The latter is known from European Russia and resembles *Krateraspis* in the elongation of the head, the shape of the forcipular coxosternite, the pattern of forcipular denticles, the number and arrangement of coxal pores, and the absence of ultimate pretarsi (see, e.g., Folkmanová and Dobroruka 1960).

#### Remarks on published morphological accounts.

Verhoeff (1930) indicated that *Tygarrupasiaticus* differs from *Krateraspismeinerti* in the clypeal areolation (a single long non-areolate plagula, with a short mid-longitudinal areolate strip, instead of two paired short plagulae), the shape of labrum (mid-piece not projecting backwards beyond the posterior margins of the labral lateral pieces), and the second maxillary pretarsi (absent). However, Verhoeff ignored the weak areolation on the central part of the clypeus and described an entire non-areolate plagula, even though recognizing a mid-longitudinal areolate strip. The putative difference in the labrum may be explained by artefacts. As for the second maxillary pretarsus, it was described and illustrated as missing in *T.asiaticus* by Verhoeff (1930), but this character was ignored in keys published later by the same author (Verhoeff 1937, 1939, 1942). Moreover, a pretarsus is recognizable in the second maxillae of the lectotype (NHRS-JONI 714; Fig. 18), while it is absent in ZMB No. 3610, which is an *Arrup* specimen originally misinterpreted by Verhoeff (1930) as a juvenile *T.asiaticus* (see above, under Remarks on nomenclatural issues).

### ﻿ Krateraspis sselivanovi

Taxon classificationAnimaliaGeophilomorphaMecistocephalidae

Titova, 1975

71D76AB4-F01B-5492-A7F0-07E7EFC19DF6

Figures 33–35 , 36–39 , 40–43

Krateraspis sselivanovi : Titova 1975: 41 (original description), 45, 46 (in key); fig. 2: 1–5A. Bonato et al. 2003: 543, 545, 546, 550, 551, 552, 577. Dyachkov 2019: 368, 373 (in key). Dyachkov 2020: 84.

#### Type locality.

“Tajikistan, Sharak” (Titova 1975) [Tajikistan, Khatlon region, Sharak village, ca. 38°16'N, 70°04'E].

#### Examined specimens.

***Paratypes***: 1 ♂, from [Tajikistan, Khatlon region, Mu’minobod district], Sharak, 10–20 [cm deep], 31.V.[19]65 (ZMMU Rc 8154); 2 ♂♂ and 2 ♀♀, from Sharak, 0–10 [cm deep], 29.V.[19]65 (ZMMU Rc 8167); 1 ♂, from Sharak, 10–20 [cm deep], 4.VI.[19]64 (ZMMU Rc 8175). ***Other material***: 3 ♀♀, from Sharak, 10–20, 20–30, 40–50 [cm deep], 15.X.[19]64 (ZMMU Rc 8153); 1 ♂ and 2 ♀♀, from Sharak, 0–10 [cm deep], 4.VI.[19]69 (ZMMU Rc 8163); 1 ♂, from Sharak, 20–40 [cm deep], 8.X.[19]65 (ZMMU Rc 8165); 1 ♂, from [unknown region] F-bad [Fayzobod village], *Hordeum*, 70–80 [cm deep], 30.VII.[19]66 (ZMMU Rc 8173); 1 ♂, from [Districts of Republican Subordination], Garm [village, ca. 39°1'N, 70°22'E], 21.VI.[19]69 (ZMMU Rc 8187).

#### Remarks on nomenclatural issues.

The type series of *K.sselivanovi* comprises 21 specimens: the holotype and 19 paratypes from Sharak, and another paratype from Faizobod (Titova 1975). These specimens are expected to be at the ZMMU, but we did not find the holotype, and the paratypes are not marked as such. Nevertheless, we detected six specimens that can be recognized as paratypes according to the locality and date reported on labels (ZMMU Rc 8154, ZMMU Rc 8167, ZMMU Rc 8175), while one specimen (ZMMU Rc 8187) can be recognized as not belonging to the type series, because it is from a locality not mentioned by Titova (1975). Instead, the date on the labels of the other eight specimens (ZMMU Rc 8153, ZMMU Rc 8163, ZMMU Rc 8165, ZMMU Rc 8173) do not fully correspond to the dates reported by Titova (1975), so it is uncertain whether they are paratypes or not.

**Figures 33–35.**
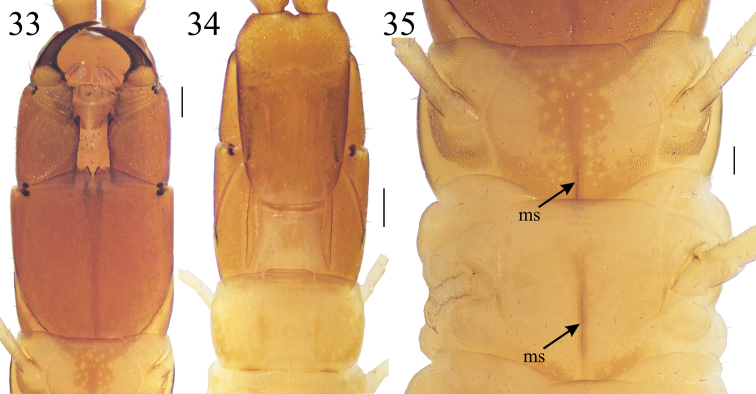
*Krateraspissselivanovi* Titova, 1975: **33, 34** head, forcipular and LBS 1 (ventr., dors.) **35**LBS 1 and 2 (ventr.). Specimen: ♂ from Sharak (ZMMU Rc 8154). Abbreviations: ms – median longitudinal sulcus. Scale bars: 0.2 mm (**33, 34**); 0.1 mm (**35**).

#### Diagnosis.

A species of *Krateraspis* with: clypeus showing the transition between marked and weak areolation at ca. 0.3–0.4 of the clypeal medial length, so that some clypeal setae are surrounded by marked areolation while other setae are surrounded by weak areolation; no spine-like sensilla on the lateral parts of the clypeus; second maxillary telopodites not distinctly surpassing the tips of the telopodites of the first maxillae; first article of the second maxillary telopodites with a distinct distal bulge on the external side; all forcipular articles with a distinct denticle with the exception of the femur; invariably 53 pairs of legs. See also Table 2.

#### Intraspecific variation.

Maximum body length: 62 mm in ♀♀ (*n* = 7) and 67 mm in ♂♂ (*n* = 8) but the largest specimens of both sexes are slightly macerated and stretched. Color (in ethanol 70%) usually yellow, with head, forcipular segment (except forcipular tergite), and antennae light brown (Figs 33, 34).

***Head*.** Anterior markedly areolate part of the clypeus extending medially for 30–40% of the total length of the clypeus (Fig. 38). Invariably eight clypeal setae: 2–4 setae on the markedly areolate part, 2–4 setae located on the border between the markedly areolate part and the weakly areolate part, and two setae on the weakly areolate one; spine-like sensilla on the clypeal lateral parts always absent. Each mandible (Fig. 39) usually with six lamellae, with 5–7 teeth in each lamella. Second maxillae (Fig. 37): 1^st^ article invariably with a distinct distal bulge on the external side; distal part of 2^nd^ article usually with two or three setae, distal part of 3^rd^ article with numerous setae.

**Figures 36–39.**
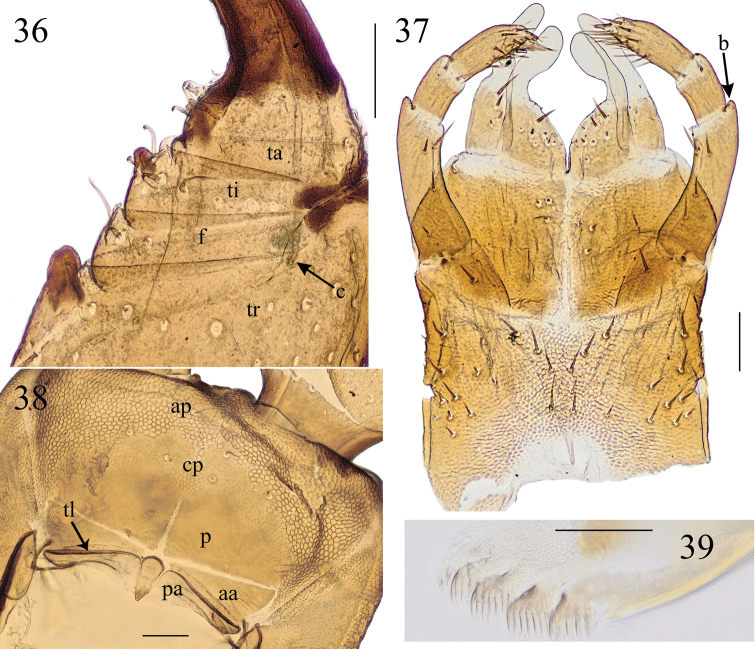
*Krateraspissselivanovi* Titova, 1975: **36** left forcipule (ventr.) **37** maxillary complex (ventr.) **38** clypeus and labrum (ventr.) **39** left mandible (lateral view). Specimens: **36, 39** ♂ from Fayzobod (ZMMU Rc 8173) **37, 38** ♂ from Sharak (ZMMU Rc 8165). Abbreviations: aa – anterior ala, ap – markedly areolate anterior part of clypeus, b – bulge, c – calyx of poison gland, cp – central part of clypeus with distinct but fainter areolation, f – femur, p – plagula, pa – posterior ala, ta – tarsungulum, ti – tibia, tl – transverse thickened line, tr – trochanteroprefemur. Scale bars: 0.1 mm (**36–38**); 0.05 mm (**39**).

***Forcipular segment*.** Tergite usually partially covered by the tergite 1. Forcipules, when closed, usually reaching the anterior margin of the cephalic plate (Figs 33, 34). Trochanteroprefemur, tibia and tarsungulum with denticles, while femur without denticle (Figs 33, 36), with the single exception of a specimen with a denticle on the right femur (however collected together with specimens with usual morphology in the sample ZMMU Rc 8163). Worth noting is that an analogous case of asymmetry has been detected in a specimen of *K.meinerti*, where a denticle has been recognized on one femur but not in the other femur (see above). The distal denticle of trochanteroprefemur usually larger than both denticles on the tibia and tarsungulum (Fig. 36). Calyx of poison gland usually reaching the trochanterophefemur in both sexes.

**Figures 40–43.**
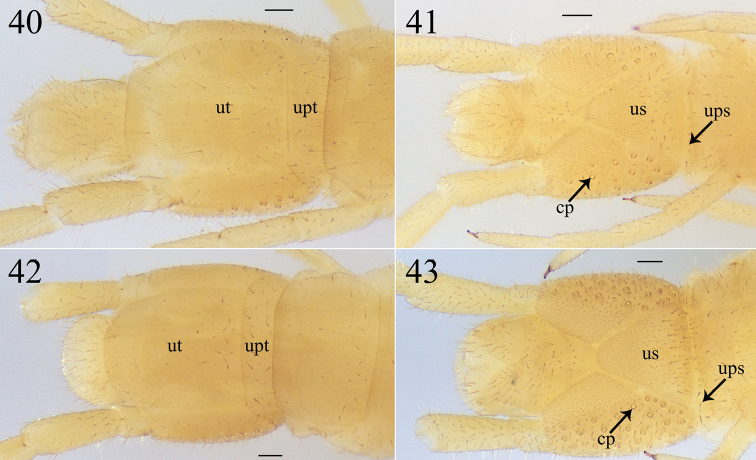
*Krateraspissselivanovi* Titova, 1975: **40, 41** ultimate LBS and postpedal segments of ♂ (dors., ventr.) **42, 43** ultimate LBS and postpedal segments of ♀ (dors., ventr.). Specimens from Sharak: **40, 41** ♂ (ZMMU Rc 8154) **42, 43** ♀ (ZMMU Rc 8153). Abbreviations: cp – coxopleural pores, ups – presternite of ultimate LBS, upt – pretergite of ultimate LBS, us – metasternite of ultimate LBS, ut – tergite of ultimate LBS. Scale bars: 0.1 mm.

***Leg-bearing segments*.** Invariably 53 pairs of legs in all examined specimens. Worth noting is that *K.meinerti* has invariably 45 pairs of legs and the difference of eight pairs between the two species corresponds to a putative evolutionary change that have repeatedly occurred in the Mecistocephalidae (Bonato et al. 2003).

***Ultimate leg-bearing segment*.** Almost similar in both sexes (Figs 40–43): metasternite subtriangular, its length to width ratio varying between 0.9 and 1.0, and the anterior margin 4–5 × wider than the posterior one; up to 20 pores on each coxopleuron in ♂♂, and up to 50 pores in ♀♀; legs slender and densely setose, without pretarsus.

***Postpedal segments*.** Densely setose in both sexes (Figs 40–43). Male gonopods bi-articulate, narrow, and separated by a conic projection in between (Fig. 41). Female gonopods bi-articulate, subtriangular, and touching each other at their bases (Fig. 43).

#### Distribution.

Recorded from three localities in the western offshoots of Pamir Mts (Fig. 1), all in Tajikistan (Khatlon region and Districts of Republican Subordination) (Titova 1975; present records).

## Supplementary Material

XML Treatment for Krateraspis

XML Treatment for Krateraspis meinerti

XML Treatment for Krateraspis sselivanovi
